# The Balance-Scale Task Revisited: A Comparison of Statistical Models for Rule-Based and Information-Integration Theories of Proportional Reasoning

**DOI:** 10.1371/journal.pone.0136449

**Published:** 2015-10-27

**Authors:** Abe D. Hofman, Ingmar Visser, Brenda R. J. Jansen, Han L. J. van der Maas

**Affiliations:** Department of Psychology, University of Amsterdam, Weesperplein 4, 1018 XA, Amsterdam, The Netherlands; Ghent University, BELGIUM

## Abstract

We propose and test three statistical models for the analysis of children’s responses to the balance scale task, a seminal task to study proportional reasoning. We use a latent class modelling approach to formulate a rule-based latent class model (RB LCM) following from a rule-based perspective on proportional reasoning and a new statistical model, the Weighted Sum Model, following from an information-integration approach. Moreover, a hybrid LCM using item covariates is proposed, combining aspects of both a rule-based and information-integration perspective. These models are applied to two different datasets, a standard paper-and-pencil test dataset (N = 779), and a dataset collected within an online learning environment that included direct feedback, time-pressure, and a reward system (N = 808). For the paper-and-pencil dataset the RB LCM resulted in the best fit, whereas for the online dataset the hybrid LCM provided the best fit. The standard paper-and-pencil dataset yielded more evidence for distinct solution rules than the online data set in which quantitative item characteristics are more prominent in determining responses. These results shed new light on the discussion on sequential rule-based and information-integration perspectives of cognitive development.

## Introduction

Two types of cognitive processing are often considered, and fiercely debated, in theoretical discussions of cognitive development: sequential rule-based processes (RB) versus information-integration (InI) based processes. These two types of processing are also contrasted in other areas in (cognitive) psychology. For example, in the study of information-integration in category learning [1] and in the study of explicit and implicit learning [2]. Moreover, Pothos [3] provides a discussion of the rules versus similarity distinction in cognition, and Kahneman [4] provides an broad overview and examples of dual route models, explicit versus implicit, in psychology.

In the study of cognitive development the balance-scale task [5] is the primary battlefield for this debate and it is the focus of this article. Recent publications [6–8] attest that this debate is still very much alive. Proponent of the RB perspective, initiated by Klahr [9] and Siegler [10], state that the cognitive process consists in the sequential comparison of different features of the stimulus. Cognitive development is described as discontinuous jumps between stages characterized by qualitatively different rules, that correspond to the consideration of different stimulus features in different combinations. With age, children acquire new insights that result in the use of more complex rules [11, 12]. From the InI perspective, cognitive processing is based on integrating different features of the stimulus before making a decision [13, 14]. Knowledge in this perspective is considered graded and implicit in nature, and development is viewed as due to changes in the implicit weights of each dimension [7, 15].

The cognitive processes used by children on the balance-scale task are especially interesting because their development spans a long period of time. Young children demonstrate interesting types of (erroneous) thinking, and many adults fail to use proportional reasoning to answer balance scale problems correctly. Also, over age, a mixture of developmental patterns seems to occur, ranging from sudden transitions to continuous change (see for example Jansen and Van der Maas [12]).

Many researchers developed computational models to investigate learning and development on the balance-scale task. Computational models from different research traditions have been proposed: production-rule models [9], decision-tree models [16], connectionist models [7, 15, 17–19] and ACT-R models [20]. Although the current models all adopt some characteristics of both theoretical positions, there is still no consensus on the best cognitive processes underlying children’s behavior in the balance-scale task [21].

In our view, this lack of consensus is partly due to the lack of adequate statistical models for the analysis of empirical data. Computational models such as production rule models and connectionist models cannot easily be fitted to data, and the existing models within the RB framework cannot test hypotheses following from the InI perspective. The empirical status of process models differs form traditional descriptive models, and a direct evaluation of these models is difficult since their aims are different [22]. In this paper we test empirical predictions that follow from both theoretical perspectives—discussed hereafter. Therefore we develop statistical models for the RB and InI perspective and a hybrid model that combines features of both theories. We apply these models to two different datasets, a paper-and-pencil dataset (N = 779) and a dataset collected within an online learning environment that includes direct feedback, time-pressure, and reward (N = 808).

### The Balance-Scale Task: Two Different Perspectives

**Fig 1:**
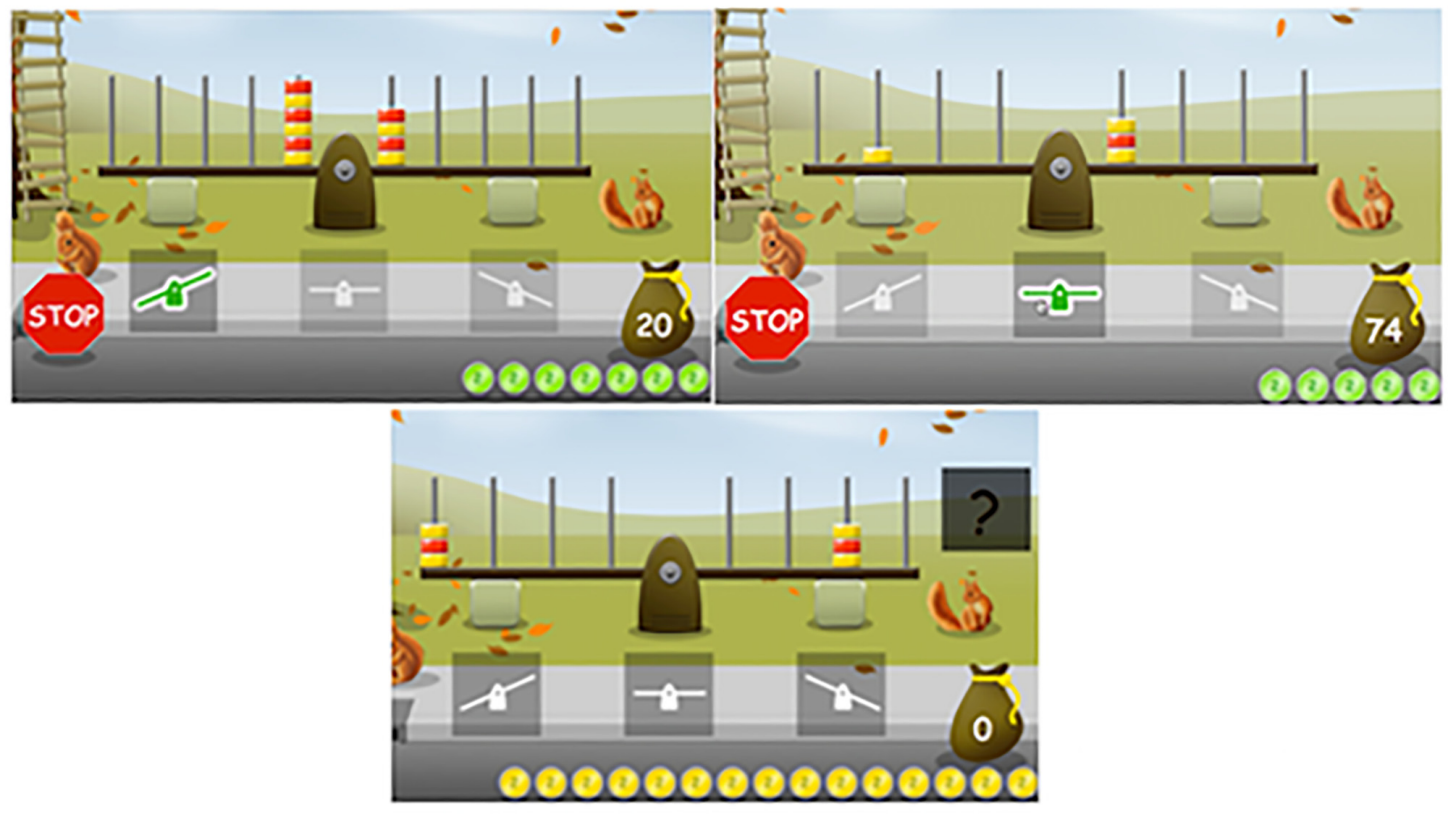
Three example items of the balance-scale task as programmed in the Math Garden (upper-left = Distance item; upper-right = Weight item, positive feedback; upper-right = Distance item; lower = Conflict-Balance-Addition item).

In the balance-scale task [5], children have to predict the movement of a balance-scale (see Fig 1), on which the number of blocks on each peg, and the distance between the blocks and the fulcrum are varied. Depending on the number of blocks and the distance between the blocks and the fulcrum on each arm, the beam tilts to one side or remains in balance. Thus, to succeed on the balance-scale task, a child has to identify the relevant task dimensions (number-of-blocks and distance) and to understand their multiplicative relation [12].

To measure proportional reasoning with the balance-scale task, Siegler [10] classified items into six item types. There are three simple item types: balance (B) items with an equal number of blocks placed at equal distances from the fulcrum; weight (W) items with a different number of blocks placed at equal distances from the fulcrum, and distance (D) items with the same number of blocks placed at different distances from the fulcrum. We also include weight-distance (WD) items, in which the largest weight is positioned at the largest distance, such that a focus on either weight (i.e. number of blocks) or distance leads to a correct answer. Next to these simple items, there are three conflict item types in which the weight and distance cues conflict: conflict-weight (CW) items, in which the scale tips to the side with the largest weight; conflict distance (CD) items, were the scale tips to the side with the largest distance and conflict-balance (CB) items where the scale stays in balance.

Using these item types Siegler [10, 23] differentiated between a postulated series of rules that children might use to solve balance-scale items. A child using Rule I will only consider the number of blocks in the prediction of the movement and disregards the distances—the number of blocks is more dominant than the distance. A child using Rule II does include the distance dimension in the prediction, but only when the number of blocks on each side of the fulcrum is equal. A child using Rule III does know that both the number-of-blocks and the distance dimension are relevant but does not know how to integrate both dimensions. A child using this rule will guess or ‘muddle through’ when both dimensions are in conflict. A child using Rule IV compares the torques on each side resulting in correct responses on all problems.

Some studies proposed alternative rules, the main example being the addition-rule (Rule III-ADD; [12, 24–26]). Children who use the addition-rule compare the sums of the number of blocks and the distance of each side of the fulcrum and predict that the side with the largest sum goes down. Detection of this rule is possible because some conflict items are solvable with the addition rule whereas others are not (see Table 1). In this study, we consider conflict items of the type conflict-balance-addition (CBA), conflict-weight-addition (CWA) and conflict-distance-addition (CDA), next to conflict-balance (CB), conflict-distance (CD) and conflict-weight (CW) items. The latter three cannot be solved with the addition rule, whereas the former can be.

**Table 1:** Siegler’s Rules on the Balance-Scale Task and the Expected Percentage (%) of a Correct Responses.

Problem Type	Rule I	Rule II	Rule III	Rule III-Add	Rule IV
Weight-Distance (WD)	100	100	100	100	100
Balance (B)	100	100	100	100	100
Weight (W)	100	100	100	100	100
Distance (D)	0	100	100	100	100
Conflict-Balance-Addition	0	0	33	100	100
Conflict-Weight-Addition	100	100	33	100	100
Conflict-Distance-Addition	0	0	33	100	100
Conflict-Balance	0	0	33	0	100
Conflict-Weight	100	100	33	0	100
Conflict-Distance	0	0	33	0	100
Rule Description	Only weight	Distance when weight is equal	Distance and weight, guess when conflict	Distance and weight, addition when conflict	Distance and weight, product when conflict

Note. Weight = Number of blocks.

In contrast to the RB perspective, according to the InI perspective children use a weighted integration of the number-of-blocks and distance between the blocks and the fulcrum, either based on a sum or a product for each side of the fulcrum and compare these integrations to select their response [13]. Either the number-of-blocks or the distance dimension is more dominant, resulting in a higher weight for one of the dimensions. In this perspective, differences between children are due to the differences in the weights that they apply to either dimension in integrating information. In the statistical extension of the connectionist models introduced in this paper, the weighted integration is only based on the sums and not the products.

### Different empirical predictions: Individual Differences and Item Characteristics

The RB and InI perspectives make different predictions about children’s behavior in the balance-scale task. Here we discuss the main differences. A first prediction concerns the characterization of individual differences between children. According to the RB perspective, children can be classified into subgroups or classes associated with qualitatively different rules. For example, Jansen and Van der Maas [26] found evidence in agreement with the RB model of Siegler [10], using latent class models. However, according to the InI perspective, these seemingly qualitative individual differences are due to quantitative differences in integration weights.

A second distinctive prediction concerns responses to different items of the same type. According to the RB perspective, the response probability is solely dependent on the item type. Items of the same item type should have equal response probabilities. This assumption of item homogeneity applies to each rule. For instance, all conflict balance items should have equal response probabilities for all users of Rule I. In the InI perspective, differences in number of blocks and distances between items of the same item type influence the response probabilities. According to Ferretti and Butterfield [24, 27, 28], children are more likely to provide correct answers when the difference between the product of the number of blocks and distance, on each side of the scale is larger. Two studies reanalyzed data of Ferretti and Butterfield [27] and concluded that this was only the case for items with extreme product differences [12, 20, 26]. Therefore, supporters of the RB perspective have argued that item homogeneity holds.

### Statistical Models: Measuring Rules vs Information Integration

As the RB and InI response mechanisms are latent (i.e., unobserved), a measurement model is required to test whether the observed patterns of responses correspond to expected responses following from the different mechanisms. The empirical detection of rules was first conducted by using rule-assessment-methodology (RAM; [10, 29]). RAM was designed to classify children to a set of a-priori defined rules, based on their observed responses instead of their verbal explanations of balance-scale answers. RAM is a two-step procedure. First, based on the set of a-priori defined rules the expected responses to the items are determined for all rules. Second, children are classified to one of the rules based on the best match between their observed responses and the expected responses following from each rule. In this classification some deviation between the observed and expected response pattern is allowed. The degree of deviation allowed depends on the item set. In the InI approach, a comparable rule-assessment method [13] is used. For some specific choice of weights, expected response patterns are calculated and children are classified as using these particular values based on their observed response pattern.

Although RAM proved to be a valuable method for studying the cognitive processes of children on the balance-scale task, is has two important disadvantages. First, RAM is not based on a statistical model, and as such does not incorporate measurement error. Hence, RAM lacks a statistical test of the fit of the classification of children to rules. As a result, it is problematic to decide on the necessity of incorporating all the rules and to compare competing rule models statistically. Second, by using a priori defined rules one risks overlooking alternative rules [30] and other response mechanisms. These limitations apply to some extent as well to the InI method of detecting integration rules used by [13].

To overcome these problems latent class analyses (LCA; see McCutcheon [31], for an introduction) were introduced in the balance-scale literature [12, 26, 32]. A latent class model (LCM) is a latent variable model, in which both the manifest (i.e., the item responses left, balance or right) and the latent (i.e., the rules) variables are categorical. Latent variable models are statistical measurement models, which allow for goodness of fit tests and statistical model comparison. It is best seen as a statistically advanced version of RAM. It is important to note that the rule model underlying RAM is in fact an instantiation of a restricted confirmatory LCM with fixed conditional probabilities [30]. Recently [21] demonstrated in a simulation study that the response probabilities of small classes (N = 20) are characterized by high standard errors. This lack of power due to small class probabilities is indeed problematic for parameter estimation in LCMs. Therefore the description and interpretation of small classes should be done with care. However, the simulation study also showed that the LCM correctly recovered the number of classes and the classification of subjects to classes, also for the small classes. To conclude, these difficulties do not outweigh the advantages of LCA over RAM [6, 8, 33, 34].

In the next section we describe the RB model and introduce a statistical InI model and a hybrid model based on predictions from both perspectives.

#### Rule-Based Model

In the LCM, both the latent variable and the responses are categorical. Participants are assigned to a latent class, associated with a distinct rule or strategy, based on their observed responses on the balance-scale items—left side down, balance or right side down. Eq 1 describes the probability of a response vector **r** in a LCM:

$$\begin{array}{c} P\left(R=r\right)=\sum_{c=1}^{C}P\left(C=c\right)\prod_{i=1}^{I}P\left(R_{i}=r_{i}|C=c\right), \end{array}$$ (1)

where *r*_*i*_ denotes the response to item *i* and *c* denotes the latent class. The LCM consists of two parts: the prior (or latent class) probabilities, *P*(*C* = *c*), describing the estimated proportion of children in a given class *c*, and the conditional response probabilities, *P*(*R*_*i*_ = *r*_*i*_∣*C* = *c*), describing the probabilities of a response to each item given a class. In our formulation, these response probabilities are estimated using a multinomial logit formulation [35]. The left response is used as the reference category resulting in two odds-ratios: left versus balance, *log*(*p*(*L*)/*p*(*B*)), and left versus right, *log*(*p*(*L*)/*p*(*R*)). The model described in Eq 1, is referred to as the exploratory model since no constraints are imposed on the response probabilities between different items.

We also consider a second LCM, in which the response probabilities between items of the same type are not allowed to vary, following the item homogeneity assumption of the RB perspective. The response probabilities can be expressed using the following logit formulation:

$$\begin{array}{c} P\left(R_{i}=r_{i}|C=c\right)=\frac{e^{\beta_{0rc}}}{1+\sum_{r=1}^{R-1}e^{\beta_{0rc}}}. \end{array}$$ (2)

The response probabilities of all items, of one item type, are modeled as a function of a general intercept *β*_0*rc*_—per odds-ratio, per item type and per class. Hence, in this model, referred to as the item homogeneity model, the response probabilities are constrained to be equal over items of each item type and each latent class. Note that the item type index is missing in Eq 2 since the model is fitted separately to data of each item type.

#### Information-Integration Model

For the InI approach a statistical model is missing. Here, we propose a new measurement model, the Weighted-Sum Model (WSM). According to the InI perspective individuals differ in two respects: a) in the dominance for either the number-of-blocks or the distance dimension and b) in the preference of balance responses. Given these two sources of individual differences the following model for the weighted-addition rule [13] is proposed:

$$\begin{array}{c} \theta_{p}=\alpha_{p}\Delta w_{i}+\left(1-\alpha_{p}\right)\Delta d_{i}, \end{array}$$ (3)

$$\begin{array}{c} \text{If}\;\theta_{p}<-C_{p}\;\text{Then}\;\text{LEFT}\\ \text{If}\;\theta_{p}>C_{p}\;\text{Then}\;\text{RIGHT},\;\text{Else}\;\text{BALANCE}, \end{array}$$

where *α*_*p*_ expresses the persons dominance for either the number-of-blocks (*α*_*p*_ >.5) or distance (*α*_*p*_ < .5) dimension, and Δ*w*_*i*_ and Δ*d*_*i*_ are defined as respectively the difference between the number of blocks (weights) and distance on both sides. Based on *θ*_*p*_ and a personal threshold, *C*_*p*_, the observed responses are derived. *C*_*p*_ serves as a boundary between responding either left or right (∣*θ*∣ > *C*_*p*_) or balance (∣*θ*∣ < *C*_*p*_). A high *C*_*p*_ implies a strong preference for the balance response. The parameters *α*_*p*_ and *C*_*p*_ are estimated per child, based on the likelihood-function of the model (see S1 Text for a detailed description of the estimation procedure). Since this statistical model is estimated per child, no distributional assumptions about the model parameters are required. According to the InI theory, differences between children are gradual and the distributions of *α*_*p*_ and *C*_*p*_ are assumed to be unimodal. A bi- or multimodal distribution of these parameters provides support for a mixture distribution representing qualitative differences between children, thereby resulting in a hybrid WSM.

#### Hybrid Models

Furthermore, to bridge the gap between the RB and InI perspective, we extend the item homogeneity LCM with item covariates [36] based on continuous item characteristics. This extension provides a formal measurement of the effect of quantitative item characteristics, such as the product-difference, on the response probabilities, combining the qualitative differences that follow from a RB perspective with quantitative item effects following from an InI perspective.

$$\begin{array}{c} P\left(R_{i}=r_{i}|C=c\right)=\frac{e^{\beta_{0rc}+\beta_{{}_{1rc}}{\text{x}}_{i}}}{1+\sum_{r=1}^{R-1}e^{\beta_{0rc}+\beta_{{}_{1rc}}{\text{x}}_{i}}}, \end{array}$$ (4)

In this LCM, the item heterogeneity model, a slope parameter *β*_1*rc*_ is included allowing for differences in the response probabilities within items of the same item type based on some item characteristic *x*_*i*_. We focus on the most often used characteristic, the product-difference (PD), the differences between the product of the number of weights and the distance on each side of the fulcrum. To conclude, we present three measurement models: a LCM following from the RB perspective, a WSM following from an InI perspective and a hybrid LCM that combines both RB and InI effects.

## Method

### Participants

The paper-and-pencil version of the balance-scale task was administered to 805 children. Responses to the first block and responses from children that did not understand the task or with missing responses (N = 26; hereafter the paper of Jansen and Van der Maas [12] is referred to as JM) were discarded. On average children needed 10 minutes to complete the test (20 seconds per item). Further details on this data set can be found in JM.

The Math Garden data set consists of data of 808 children who completed at least five blocks during the data collection period (between 2011-06-10 and 2011-08-12). In the Math Garden children practiced either during school or outside school hours, resulting in large differences in both the number of items made and in the amount of time spent playing the balance-scale game. On average these five blocks were completed within 8 days (SD = 10.5, range = 0–54). The responses on items of the first block were discarded since children had to get acquainted to the task. Table 2 shows the distribution of age of both the paper-and-pencil and the Math Garden dataset. Note that older children are somewhat underrepresented in the Math Garden dataset compared to the paper-and-pencil dataset.

**Table 2:** Distribution of Age for the Paper-and-Pencil and Math Garden dataset.

age in years:	< 6.00	6.00–7.99	8.00–9.99	10.00–11.99	12.00–13.99	14.00–15.99	> 16.00
Paper-and-Pencil	1	63	148	171	146	147	93
Math Garden	15	209	281	186	41	14	0

### Materials

#### Paper-and-Pencil

The paper-and-pencil version of the balance-scale task consisted of five items of the types W, D, CW, CDA and CBA (see S2 Text for the item characteristics). Before administration of the task, the experimenter explained that the pegs were placed at equal distances, that all the weights had the same weight, and showed that a pin prevented the scale from tipping. Subsequently, three example items were presented to familiarize the children with the format of the test.

#### Math Garden

In the balance-scale game, children are asked to predict what would happen if the blocks under the balance are removed (see Fig 1). The three answer options are displayed below the item. The Math Garden game differs in three respects from the standard paper and pencil test. First, items are presented with a time-limit of twenty seconds. Second, children receive feedback on the accuracy of their response directly after responding. Third, children are rewarded for correct responses and are punished for incorrect responses. The time-limit/pressure is an inherent aspect of the feedback system where size of reward/punishment is positively related to speed [37]. If a child has no clue of an answer he or she may press the question mark button. These task elements are designed to keep the task challenging, and enable learning through feedback (see [38], for an extended description of the Math Garden system and its rationale).

The original item set consisted of 260 items, divided in twenty blocks of thirteen items of different types. Ten item types are presented in Table 1. The remaining item types were items with weights on multiple pegs on one or two arms of the scale. We analyze responses to the four D, CW, CDA and CBA items to increase comparability with the paper-and-pencil and the Math Garden dataset (see S2 Text for the characteristics). In both datasets, for all item types, except CBA items, the quantitative item characteristic of interest was the product-difference. For CBA items we use weight-difference as an alternative (for CBA items the product-difference is zero by definition since the weight- and distance-differences are the same). Although the items were not explicitly constructed to test a quantitative effect, they exhibit sufficient variation in this item characteristic. For both datasets the responses were recoded such that the correct response is the left response for D, CW and CDA items, and such that the largest amount of pegs resides on the left side of the fulcrum for CBA items.

### Model Estimation and Comparison

Following the approach of JM, we applied LCA in two consecutive steps. First, the responses per item type were investigated. The number of latent classes was determined (investigating qualitative individual differences) with exploratory LCA (the exploratory model). Thereafter, parameter restrictions, formulated in the item heterogeneity model and the item homogeneity model, were sequentially tested. Second, building on the results of this fitting procedure per item type, response to multiple item types were analyzed with the hybrid LCM (item heterogeneity model; formulated in Eq 3). This approach reduces the sparse data problem in LCA when analyzing a large set of variables since it limits the number of estimated parameters compared to exploratory model. Hence the power to detect different classes increases. Third, this item heterogeneity model—the hybrid LCM—is compared with the item homogeneity model—the rule-based model.

For the LCM including all responses, we analyzed the posterior probabilities, *P*(*C* = *c*∣**R** = **r**). These probabilities—based on the observed responses of a person and the estimated prior and conditional response probabilities—indicate the classification probabilities of a person to each class. The probabilities are related to the homogeneity of responses of subjects belonging to a certain class and the class separation [39]. A high (maximum is one) posterior probability implies that the observed response pattern of a subject is well described by the estimated response probabilities of a latent class. A value of one divided by the number of classes indicates that the observed responses pattern cannot be clearly assigned to any latent class. The average (and standard deviation) of the posterior probabilities over subjects assigned to each class is presented. A high mean indicates that subjects can be clearly assigned to this class compared to the other classes.

All RB and hybrid models were estimated with the depmixS4 package [40] in R [41]. For stable model estimation we scaled the product-difference, per item type, such that the mean equals zero. Twenty replications were used with random starting values to prevent solutions based on local optima. All presented models were stable. We used the Bayesian Information Criterion (BIC) [42] for model selection since this fit measure provides a good balance between goodness-of-fit and parsimony [43]. In addition, BIC-weights, *P*(*BIC*), are presented to facilitate the interpretation of BIC differences. BIC-weights are transformed values of the BIC differences to a probability scale representing the probability of each model being the best model given the data and the set of candidate models [44].

For the estimation of the WSM only responses to the conflict items were analyzed since simple items can be solved without the integration of the two dimensions and therefore do not discriminate between differences in the integration strategy [13].

## Results

To investigate whether children in the Math Garden version of the balance-scale task understood the task we first fitted the exploratory model to WD items. All children should succeed on these items. The LCM with two classes showed the best fit (see Table B in S3 Text). Responses of children assigned to the class with high probabilities (N = 667) of a correct response (on average 93% correct) were included in further analyses. Of the selected children, 603 played the task before the start of the study, and made on average 800 items (SD = 965, range = 1–7695). Subjects with missing responses were only excluded if the missing response corresponded to the investigated item type, resulting in a different number of children for each analysis. 566 children responded to all selected items. In the next section we compare the results of the exploratory model, item heterogeneity model and the item homogeneity model, per item type in the two datasets.

### LCM per Item Type

#### Distance

**Fig 2:**
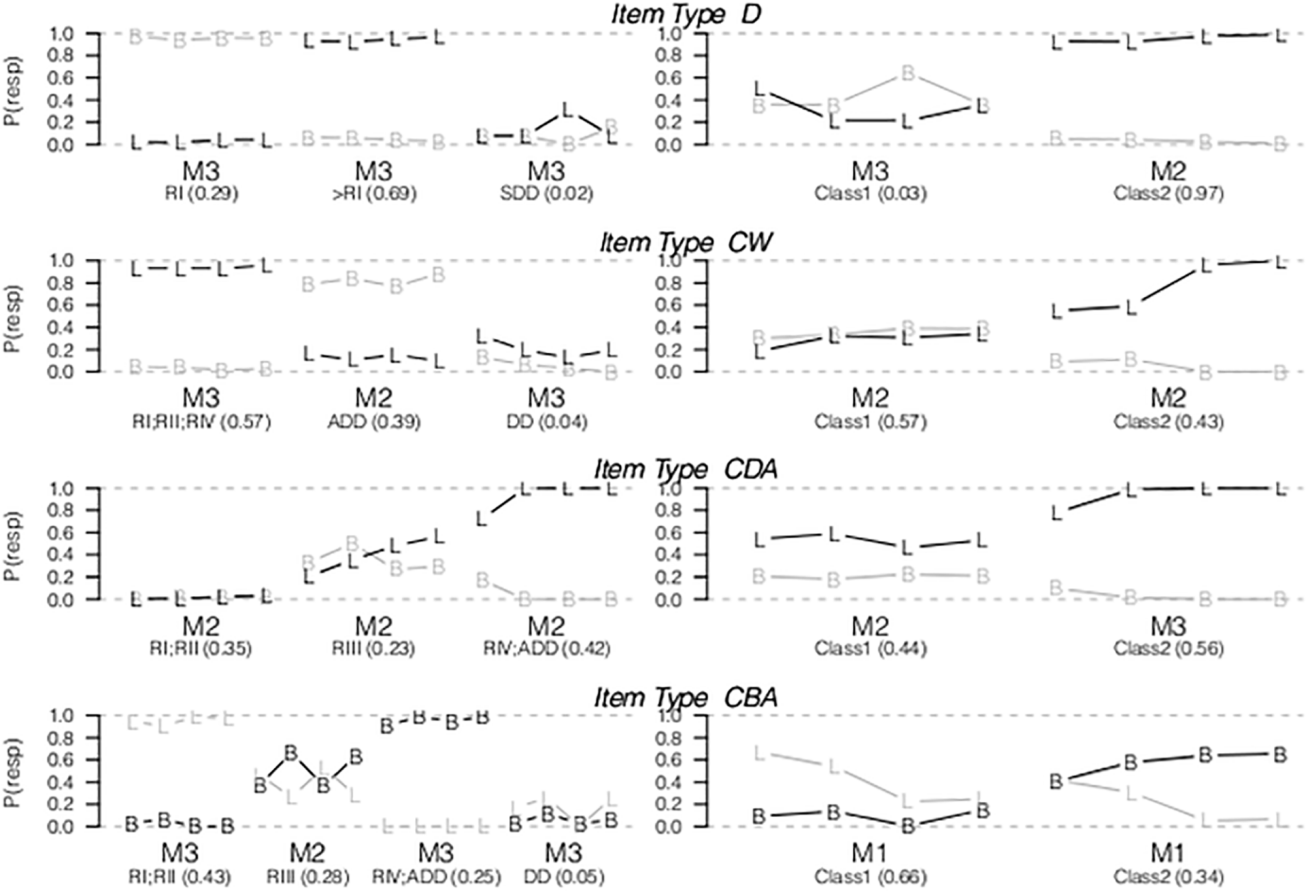
The observed response probabilities (y-axis) of the left (L) and balance (B) response of respectively the paper-and-pencil dataset (left panels) and the Math Garden dataset (right panels), ordered on the product-difference or weight-difference for CBA items (x-axis). M1 (exploratory model), M2 (item heterogeneity model) and M3 (item homogeneity model) indicate which model provided the best fit. The x-axis labels show the class description of JM for the paper-and-pencil dataset, and the prior probabilities between brackets. The Small-Distance-Down, Distance-Dominant and Addition Rules are abbreviated as SDD, DD, and ADD.

For the JM dataset, the three class item homogeneity model was the best fitting model for D items. The observed response probabilities of each class are presented in Fig 2. The three classes resembled respectively children that provided balance responses (Rule I), provided the correct left responses (Rule II or more advance strategies), or predicted that the side with smallest distance goes down. See Table A in S3 Text for the goodness-of-fit statistics of all models. Although JM concluded that the responses of children were best described by four qualitatively different rules, the BIC indicated that the three-class model showed the best fit for the paper-and-pencil dataset. This difference results from a different model specification. JM analyzed direct response probabilities, whereas we used a logit transformation of the odds ratios (see Methods section). As a result some conditional probabilities of JM were zero and therefore these parameters did not contribute to the model fit, which is not possible in the logit model specification. For the Math Garden dataset, two classes were needed to describe the observed responses. The first class showed an average probability of the correct left response of .36, and the product-difference did not relate to the response probabilities (item homogeneity model). This class is described as guessing behavior. The second class showed a high probability of the correct response indicating that these children use a more advanced rule than Rule I. Furthermore, for this class the probability of a correct response was higher for items with a large product-difference (item heterogeneity model) indicated by an increase in the left-right and left-balance odds ratio. The first latent class found by JM, described as Rule I, was not found in the Math Garden dataset.

#### Conflict-Weight

For the JM dataset, the three-class model showed the best fit. These latent classes resembled the classes found in JM, described as: a class of children with near perfect responses (Rule I, Rule II or Rule IV), a class of children using the addition rule and a class of children that perceived the distance dimension as the dominant dimension (DD). For each latent class the item homogeneity model resulted in the best fit, i.e., item responses were homogeneous across different product-difference values. For the Math Garden dataset, the two-class model showed the best fit. Moreover, the item heterogeneity model fitted better than the exploratory model and the item homogeneity model. The first class showed a low probability of the correct response. The second class showed an overall high probability of the correct response corresponding to Rule I, Rule II or Rule IV (the first class in the paper-and-pencil dataset). This indicated that children in the second class perceived the number of blocks as dominant whereas children in the first class perceived distance as dominant. The positive relation between the response probabilities and the product-difference of the item showed that responses of children improved with increasing product-differences.

#### Conflict-Distance-Addition

The LCM for the JM dataset resembled the results of JM, and consisted of three classes resembling Rule I or Rule I, Rule III and Rule IV or an addition rule, respectively. Moreover, the item heterogeneity model resulted in the best fit for each latent class. These results correspond to the results of JM, since they also found that the response probabilities of CDA items could not be constrained over items that differed with respect to the product-difference. Even for children using Rule I or Rule II (class 1) the probability of the correct response increased as a function of the product-difference. In the Math Garden dataset, the two-class model showed the best fit. In the first class the item heterogeneity model and in the second class the item homogeneity model resulted in the best fit. The first class showed an average probability of the correct response of .5. Children in the second class showed a probability of the correct response of .9.

#### Conflict-Balance-Addition

In the JM dataset, the four-class model showed the best fit, resembling the results of JM. Children in the first class had a high probability of the left response (the side with the largest number of blocks), resembling Rule I or Rule II. Moreover, the LCM with a negative effect of the weight-difference in the second latent class (Rule III) resulted in the best fit. For children in this class, the probability of a correct response was smaller for items with a large differences in the number of blocks between the sides of the fulcrum. The response probabilities of the third class are described by JM as produced by children who use Rule IV or the addition rule. For the Math Garden dataset, the two-class exploratory model showed the best fit. Hence, the variation in the observed response probabilities cannot be explained by the weight-differences of the items. Also, the LCM did not reveal a class of children with a high performance on CBA items.

#### Conclusions

The LCMs based on the paper-and-pencil dataset replicated, in general, the class structure found by JM. In contrast, the models based on the Math Garden dataset deviated in number and description of the classes. In eight out of thirteen latent classes in the models for the paper-and-pencil dataset, the responses of children were best described by the rule-based item homogeneity model, but this model was the best model in only two out of eight latent classes of the models for the Math Garden dataset. In the majority of the classes in Math Garden dataset the item heterogeneity model appeared to be the best model.

### Mix of Item Types

The following analyses concerned responses to multiple item types. We estimated a second set of hybrid and RB LCMs and applied the WSM to a selection of items of different item types.

#### LCM

In the LCM it is assumed that the responses to items of the same type can be modeled as repeated measures, only allowing variations as a function of the product- or weight-difference of the items. This assumption is not met for the item types where the exploratory model showed the best fit in the previous analysis (see results of the CBA items in the Math Garden dataset). Therefore, in the Math Garden dataset responses to all D, CW, and CDA items and only the last CBA item were selected and in the paper-and-pencil dataset all responses were selected.

#### Paper-and-Pencil Dataset

We estimated LCMs with one to ten latent classes. As can be seen in Table 3, the BIC and *p*(BIC) indicated that the LCM with nine classes showed the best fit. Furthermore, the RB LCM resulted in a better fit than the hybrid LCM (see Table 3).

**Table 3:** Fit Results LCM mix of item types.

Paper-and-Pencil Dataset						Math Garden Dataset					
Model	NC	NPar	BIC	1:*p*(BIC)	2:*p*(BIC)	Model	NC	NPar	BIC	1:*p*(BIC)	2:*p*(BIC)
Hybrid	6	101	12357	<.001		Hybrid	2	29	9541	<.001	
Hybrid	7	118	12343	<.001		Hybrid	3	44	9465	.108	
Hybrid	8	135	12336	<.001		Hybrid	4	59	9461	.892	>.999
Hybrid	9	152	12315	>.999	<.001	Hybrid	5	74	9516	<.001	
Hybrid	10	169	12373	<.001		Hybrid	6	89	9565	<.001	
RB	9	88	12152		>.990	RB	4	35	9510		<.001

Note. For the paper-and-pencil (N = 779) and Math Garden (N = 566) dataset, responses of 16 and 13 items, respectively, were analyzed; NC = number of latent classes; NPar = number of parameters; *p* = BIC weight (1) for comparison of models with different number of classes and (2) for the comparison of the hybrid and RB model.

**Fig 3:**
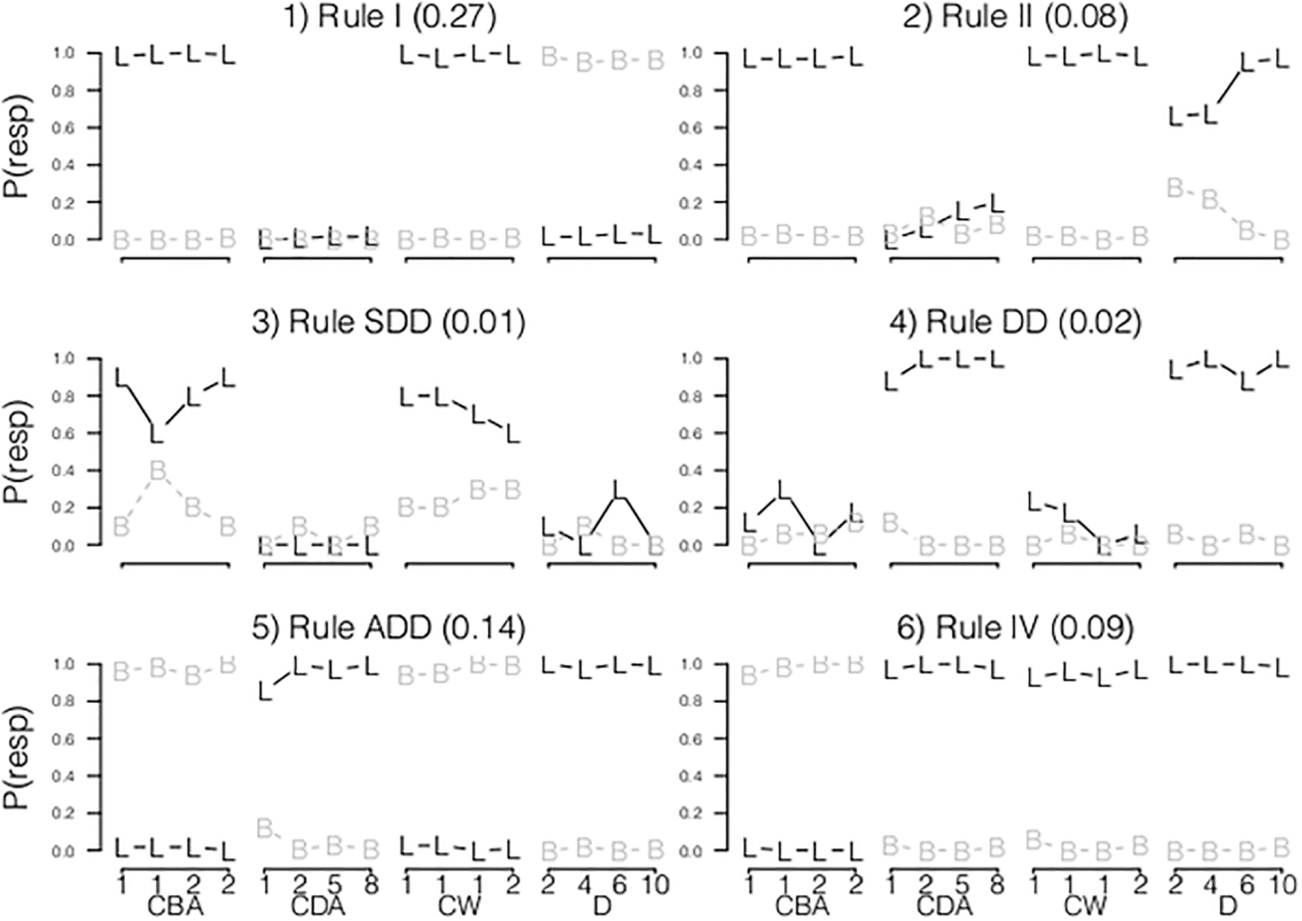
Paper-and-Pencil Dataset. The plots show per class the response probabilities, per item type ordered on the quantitative item effect (on the x-axis).

Fig 3 shows the response probabilities of the nine classes. The first six classes represented a clear Rule I, Rule II, a small-distance-down (SDD) rule, a distance-dominance (DD) rule, addition (ADD) rule and Rule IV, replicating the findings of JM. Moreover, the average person fit (the posterior probabilities of class membership) of these classes showed that subjects could be rather clearly assigned to most of these classes, respectively .95 (SD = .09), .65 (SD = .18), .77 (SD = .22), .67 (SD = .17), .65 (SD = .15) and .75 (SD = .17). The fourth class, representing the DD rule, was also found in the LCM results per item type. This class was probably not found by the analyses of a mix of item types by JM because of a lack of power. The higher power is achieved by a different item selection and the use of item covariates in the LCM. The sixth class, representing Rule IV, showed perfect performance on all items.

**Fig 4:**
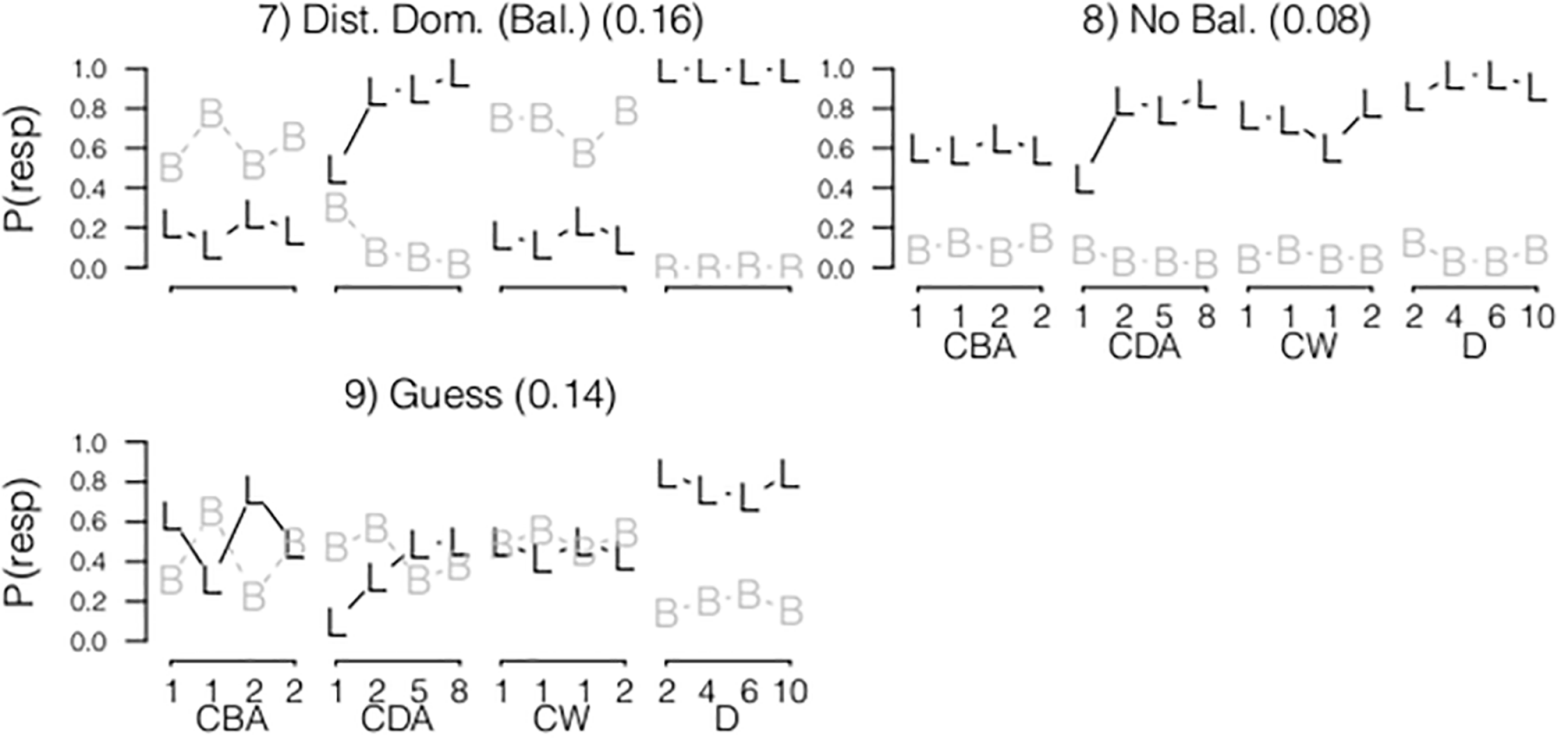
Paper-and-Pencil Dataset: Response probabilities of the LCM on a mix of item types. The plots show per class: the response probabilities, per item type ordered on the quantitative item effect (on the x-axis).

The remaining two classes in JM were interpreted by JM as either Rule III or Rule III/ADD. The current analyses led to three extra classes rather than two, probably as a result of the higher power. The posterior probabilities of the LCM showed that the classification of children to rules was rather ambiguous for these remaining classes, indicated by the high variation and the overall low fit of respectively, .56 (SD = .15), .57 (SD = .19) and .63 (SD = .19), for class 7, 8 and 9 (see Fig 4). Hence, the response probabilities cannot be reliable interpreted as governed by a distinct set of rules. Therefore, these classes are only loosely described as: a distance dominant class providing a lot of balance responses (class seven), a class providing left or right responses (eight) and a class that guessed between the left and balance response (nine).

To conclude, in general, the results of JM are replicated with the new LCM. The gained power to detect individual differences resulted in two additional classes. The person fit indicated that subjects assigned to these latter classes showed a high response variability. Hence, the response patterns were difficult to interpret and could not be ascribed to a clear set of rules.

#### Math Garden Dataset

**Fig 5:**
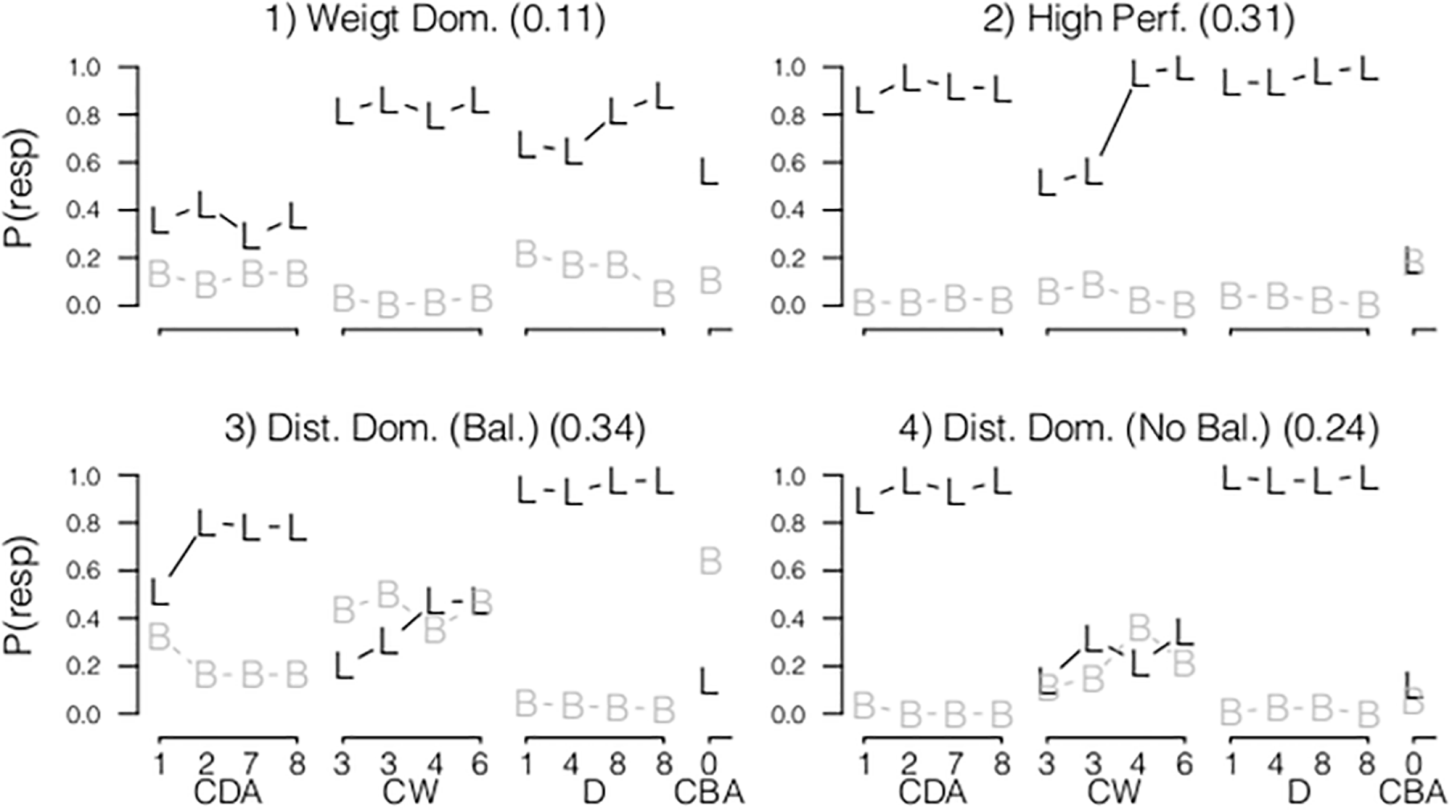
Math Garden Dataset: A description of the four classes of the LCM on a mix of item types. The response probabilities are depicted (on the y-axis), per item type ordered on the quantitative item effect (on the x-axis).

For the Math Garden dataset, the fit of the sequence of LCMs indicated that four classes were needed to describe the responses, according to the BIC (Table 3). Fig 5 provides a description of the LCM. The first class (Weight Dominant) had a high probability of the correct response on CW and a low probability on CDA items. Furthermore, the high probability of the left response on the CBA items showed that subjects perceived the number-of-blocks dimension as more dominant. These response probabilities resembled to some extent Rule II. The second class showed high performance on all item types, except on the CBA item. Again, the high probability of the right response on the CBA item indicated that children in this class perceived the distance dimension as more dominant than the number-of-blocks dimension (for CBA items the right side is the side with the largest distance). In the third class the probability of a correct response was higher on CDA items than on CW items, and highest for D items. Moreover, the high probability of the right response on the CBA item indicated that the distance dimension is perceived as dominant. The forth class mostly resembled the third class, with the addition that the response probabilities for a balance response were considerably lower on CDA, CW and CBA items compared to the third classes.

In general, Fig 5 shows that none of these classes resembled Rule I, Rule II, SDD or Rule IV, but rather resembled variations of Rule III. Also, distance-dominant classes were found that have not been reported earlier in paper-and-pencil versions of the balance-scale task. As indicated by the BIC-weight, the response probabilities depended on the product-difference of the item. The probability of the correct response is higher for items with a larger product-difference. Finally, the average posterior probabilities of the LCM, respectively .58 (SD = 23), .64 (SD = 11), .62 (SD = .19) and .59 (SD = .16) indicates that children could not be clearly ascribed to one of the four classes.

#### Conclusion

A comparison of the results of the LCM of both datasets show that large differences are present in the response mechanism. This is alluded by the better fit of the hybrid LCM in the Math Garden and the rule-based LCM in the paper-and-pencil dataset. Moreover, in the paper-and-pencil dataset the majority of children could be clearly ascribed to latent classes representing qualitative different rules, earlier described by Siegler [23] and JM [12]. In the Math Garden dataset the four classes did not resemble any earlier found strategies. Also, the overall lower posterior probabilities showed that differences between the children were more of a quantitative nature when tested in the Math Garden.

#### Age and Practice Effects

**Fig 6:**
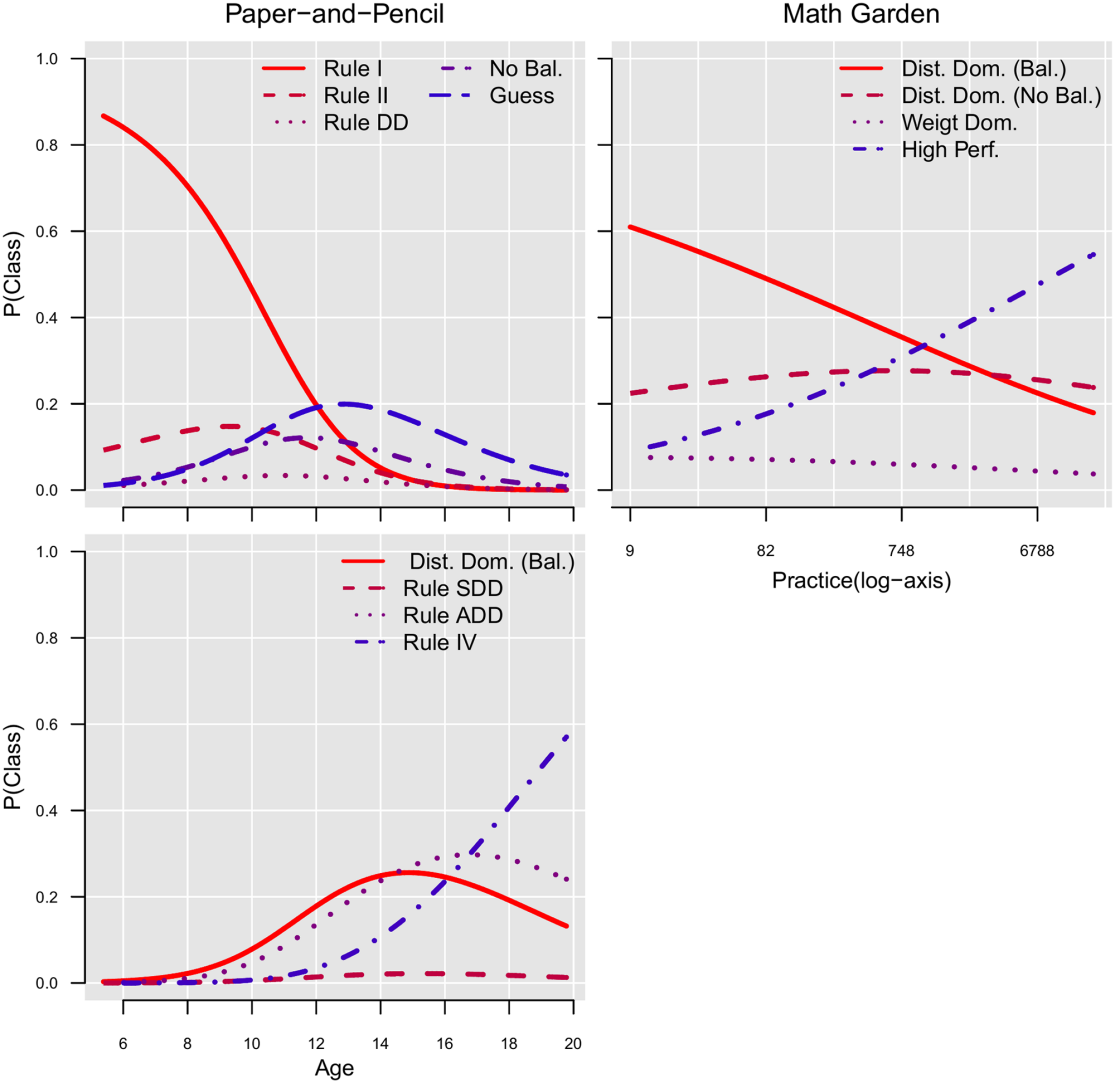
The effects of Age in the Paper-and-Pencil data (left) and practice in the Math Garden data (right) on the class membership of the latent class models.

JM already showed that large age differences are present between children classified to different classes in the paper-and-pen dataset. Using the latent class models introduced in the current paper, we investigated the relation between the dependent variable class membership in the best fitting latent class model (nine classes), and the independent variable age using multinomial regression models. Different models are compared based on the BIC. Results of the paper-and-pencil data again showed large age effects (BIC of model with and without age was respectively 2673 and 3113)). In the Math Garden dataset, age was not related to class membership (BIC of model with and without age was respectively 1140 and 1125). However, the class membership was related to the amount of practice (BIC of model with and without practice was respectively 1120 and 1125). Practice was defined as the log of the number of items made before the start of the data collection. We use the log function to transform the skewed distribution of the number of item made per child to a normal density. Fig 6 shows the predicted probability of a child being assigned to each class as a function of age for the paper-and-pencil data, and as a function of practice for the Math Garden data.

In line with the previous results, large differences are found between both analyzed datasets. In the paper-and-pencil data a clear developmental change is highlighted by the age effect (further described by JM). In the Math Garden data the developmental pattern is solely based on the amount of practice.

#### WSM

**Fig 7:**
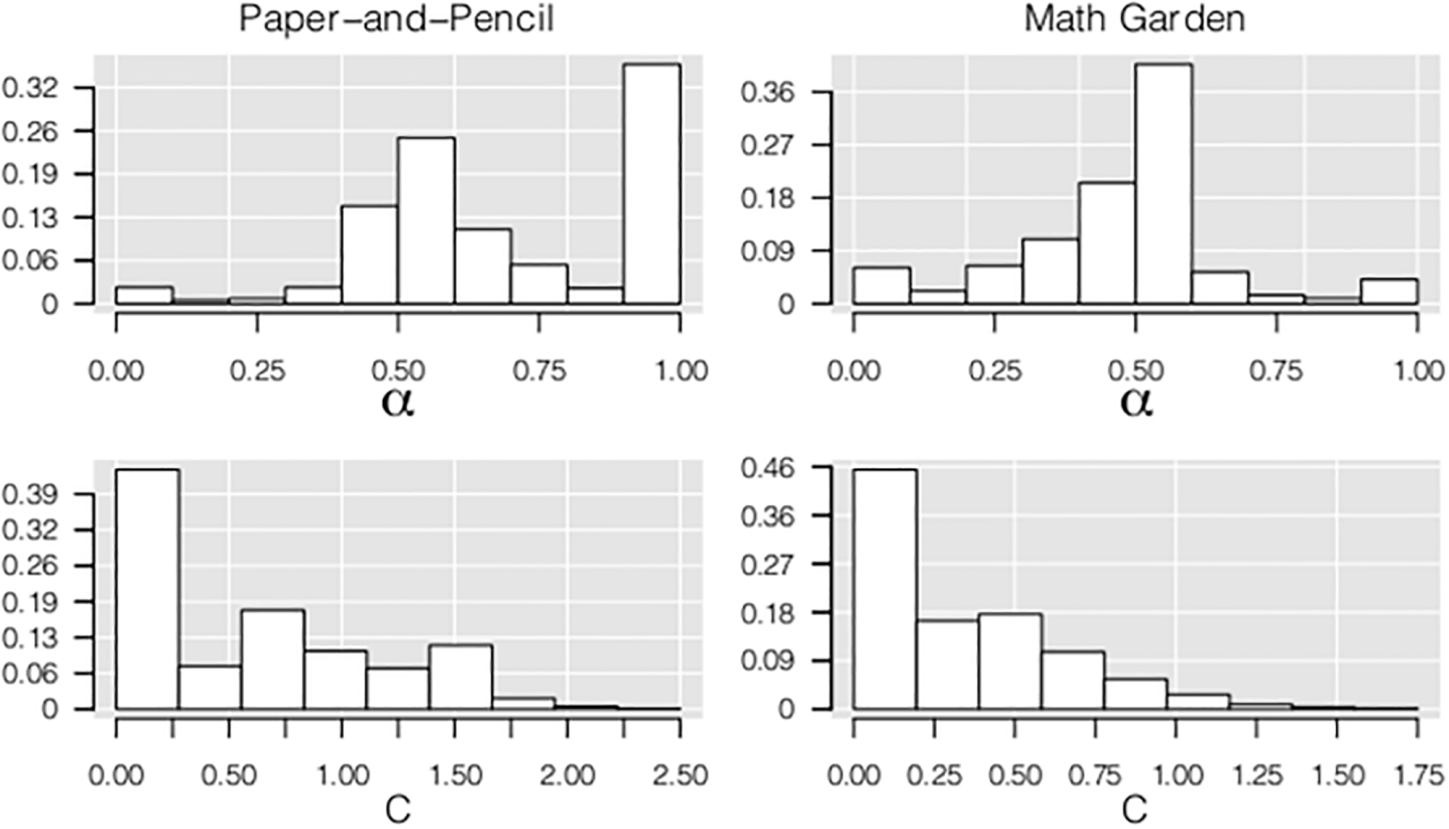
Distributions of *α*, *C* of the WSM for the paper-and-pencil and Math Garden dataset.

Fig 7 shows the distribution of the estimated *α* and *C* parameters of the WSM based on responses to the four CW, CDA and CBA items. In the paper-and-pencil dataset the distribution of *α* was clearly not unimodal, and deviated from a normal density as indicated by the Shapiro-Wilk test [45] (D = .226, p < .001). The large peak at *α* = 1 reflected that some children (N = 277, 36%) only responded to the number-of-blocks dimension, including children using Rule I and II [13]. The smaller peak at *α* = 0 indicated that only the distance dimension was reflected in the responses of 2.4% of the children (N = 19). Both values of *α* indicate that these children did not integrate the information regarding both dimensions. Furthermore, the distribution around *α* = .5 illustrated that the remaining children weighed both dimensions about equally in their responses. The distribution of *C* showed that 45% of the children already predicted that the scale would tip to a side when their integration of both dimensions resulted in a value just above zero (note that this does not mean they did not provide any balance answers).

In the Math Garden dataset, the distribution of *α* showed a different pattern—again the distribution deviated from a normal density (D = .145, p < .001). In contrast to the paper-and-pencil dataset, the peak at *α* = 1 was small (N = 23, 4.2%). The large distribution around *α* = .5 showed that the majority of the children weighted both dimensions about equally. However, also a small peak at *α* = 0 was found representing children who only took the distance-dimension into account (N = 34, 6.2%). The distribution of *C* resembled the distribution in the paper-and-pencil dataset. The majority of the children decided that the scale would tip if their outcome of the weighted integration of the differences between the arms was higher than zero.

#### Conclusion

The distribution of *α* and *C* indicated that also qualitative differences were present since differences between children cannot be described by an unimodal distribution. Moreover, as mentioned previously, a substantial group of children did not integrate information of both dimensions. Hence, a hybrid WSM model is needed to provide a description of the full range of individual differences. However, further developments of the WSM are needed to investigate this. The estimation of the WSM to responses of multiple subjects, and the formulation of the random parameters therein, should provide a test on distribution of these parameters, resulting in a formal test of the InI versus hybrid account. However, a visual inspection of the distribution of the model parameters over persons clearly indicates that a rule-based component is needed to fully explain the observed responses within the WSM framework.

## Discussion

The aim of the paper was to compare a RB and an InI perspective on the cognitive processes used by children to solve balance-scale items, using a new set of statistical models.

According to the LCM analyses aspects of the InI perspective are required to describe the Math Garden data and the CDA items in the paper-and-pencil dataset. The results of the WSM, allowing for quantitative (continuous) differences between children in the preference of the number-of-blocks or distance dimension and the preference for balance responses, indicate that quantitative and qualitative differences show up in the inspection of the distribution of the estimated parameters. Hence, results of both statistical models support a hybrid account integrating RB and InI perspectives.

Although we found additional classes in the paper-and-pencil dataset, the majority of children can be clearly assigned to one of the rules described by Siegler [10, 23] and Jansen and Van der Maas [12, 26]. None of the classes in the Math Garden dataset resembles any of these earlier proposed rules. The results indicate that children tested within Math Garden integrate the number-of-blocks and distance dimension to solve balance-scale problems. However, although some children did play the task intensively prior to this study, the LCM did not reveal any children with a perfect integration rule (RIV users). Additionally, whereas Siegler [10] stated that the number-of-blocks dimension is the dominant dimension, both the LCMs and the WSM reveal that a subset of children perceive the distance dimension as dominant.

In the Math Garden data, the response probabilities are related to differences in the product-difference between items, and to a much smaller extent in the paper-and-pencil dataset. This undercuts the conclusions by Jansen and Van der Maas [26] and Van Rijn, Van Someren and Van der Maas [20] that this item characteristic was only related to the response probabilities of items with extreme product-differences. Based on a latent-class regression modeling approach resulting in more power to detect an effect of the product-difference, our results indicate that items with a larger product-difference are easier than items with a small product-difference even for items with a reasonably small product-differences. Moreover, the magnitude of this effect differs between both datasets.

Although in both datasets a hybrid account is evident to fully explain the responses of children, differences between both datasets are present as well. In the classical paper-and-pencil version of the task, collected under the standard task demands, cognitive processes are best described by a RB perspective, with the exception of the product-difference effect that follows from a InI perspective. Testing children within the Math Garden, with direct feedback, time-pressure and a rewards system, seems to induce a different cognitive process, providing more evidence for elements of an InI perspective. Where the debate between the RB and InI perspectives in the field of proportional reasoning is concerned with the underlying mechanisms of one cognitive process (or a single response mechanism), the results of this study indicate that the characteristics hereof might depend on the task demands. Positioning the findings based on the Math Garden data alongside the findings of the paper-and-pencil dataset suggests that different response mechanisms are at play. This result sheds new light on the debate of RB and InI perspective in the balance-scale literature.

This study was not designed to investigate and isolate the effect of task demands. Also, both age and amount of experience with the task of the tested children differs between both datasets, and have a different relation to the latent classes. Further research is needed to determine which factors influence the response mechanism of children. However, it is surprising that so far, the predictions following from both rule-based and information-integration perspectives on children’s knowledge on the balance-scale task, have mainly been tested with only one type of empirical data: responses to a paper-and-pencil test and the computer analogue thereof [12, 26, 46]. This is even more surprising since Ferretti [27] already showed that rule assignments differ when children are asked to rebuild one side of the scale instead of predicting the movement.

In other fields of cognitive psychology it is known that task demands influence the type of cognitive processes (or response mechanisms) that are activated or learned. For example, in category learning, differences in the type of task result in the use of qualitatively distinct learning systems [47], and task demands such as time-pressure and feedback have different effects on these distinct learning systems [48, 49]. Maddox, Bohil and Ing [50] show that the performance on a rule-based learning task is impaired when subjects have a short period to process the feedback after a response, while this manipulation did not affect the performance of subjects using information-integration (or similarity) based learning processes.

Therefore, we argue that the differences between the results of both datasets in the present study, are best understood by relating these differences to the differences in the task demands under which children are tested. Based on the described literature, it is expected that the influence of feedback, time-pressure and/or a reward system promotes the usage of different processes. This possible influence of task demands on the response mechanism and an appeal for the integration of RB and InI perspectives in a model of development is already made by Fischer [51] [p.626]: “under certain conditions of observation and degrees of abstraction, universal stages of cognitive organization can be observed; under others, important individual differences in developmental sequences occur.” They conclude that: “What is needed is a view fully grounded in the fact that cognitive development appears diverse under some observational conditions and universal under others.” This is also alluded to by McClelland [15], since he states that rule-like behavior can be induced by different testing situations.

To make the RB perspective compatible with the current results, at least one of the available response mechanisms should be of a more quantitative (similarity-based) nature. The description of Rule III [10] production model provides such a possibility. Siegler describes children using Rule III as “muddling” through. This strategy could include a mixture of implicit information integration strategies and a preference could be present for either the number-of-blocks or the distance dimension. Moreover, for these children the responses could be based on quantitative item characteristics resulting in the presence of for example a relation between the product-difference and the response probabilities.

To make the InI approach compatible with the current results, it would be necessary to incorporate some qualitative rule-based effects, as found in the LCMs of both the paper-and-pencil and Math Garden dataset. The work of Dandurand [21, 52] already combines RB effects in an InI approach by including an external learning module in which the model is ‘taught’ RIV—the correct rule where the difference is calculated between multiplication of the weights and distance on each side of the fulcrum. This approach is based on the assumption that children might also learn this rule in an educational setting from instruction instead of from their own experience, which makes it an explicit rule. Such an interpretation of RIV performance fits very well in a rule-based approach. Furthermore, Schaprio and McClelland [7] also propose a combination of RB and InI processes. They state that: “It is possible that the best account will involve a mixture of explicit and implicit strategies.”

To describe the cognitive processes of children used on a proportional reasoning task like the balance-scale task, a model is required that (1) incorporates both a RB and a InI account and (2) specifies in what conditions the behavior is caused by which account. Hybrid models with components relying on rule-based and similarity-based processing of items have become the norm in modeling categorization learning, for example COVIS [53] and Atrium [54]. These models can serve as a valuable starting point for including multiple response modes based on different response mechanisms for development of proportional reasoning in general and balance-scale learning specifically under different task demands.

## Supporting Information

S1 Text (PDF)

S2 Text (PDF)

S3 Text (PDF)
