# Supplementary material for: The Balance-Scale Task Revisited: A Comparison of Statistical Models for Rule-Based and Information-Integration Theories of Proportional Reasoning
Source: PLoS One. 2015 Oct 27;10(10):e0136449. doi: 10.1371/journal.pone.0136449 (PMC4623502; doi:10.1371/journal.pone.0136449)
Supplement: S1 Text — (PDF) [file pone.0136449.s001.pdf]

S1 Text

The estimation of the weighted-sum rule is based on the optimization of the following set of equations, for each subject. First, the person vector θ_p is based on the implicit weight for the number-of-blocks ($\alpha_p > .5$) or distance ($\alpha_p < .5$) dimension and on the characteristics of the item set:

$$\begin{aligned}\boldsymbol{\theta_p} &= (\alpha_p \mathrm{wl} + (1-\alpha_p)\mathrm{dl}) - (\alpha_p \mathrm{wr} + (1-\alpha_p)\mathrm{dr}) \\ &= \alpha_p \Delta w_i + (1-\alpha_p)\Delta d_i.\end{aligned}$$

Based on the scaled $\boldsymbol{\theta_p}$ ($\sigma = 1$), and assuming a normal density to express the response probabilities, the log-likelihood of the response vector can be expressed as follows (person subscripts are dropped):

$$\begin{aligned}\log L(\boldsymbol{\theta}, C) = \sum_{i=1}^{I} (R_i = l) \log \int_{-\infty}^{-C-\theta_i} \frac{1}{\sqrt{2\pi}} e^{\frac{-(x-\theta_i)}{4}} dx + \\ (R_i = b) \log \int_{\infty}^{C-\theta_i} \frac{1}{\sqrt{2\pi}} e^{\frac{-(x-\theta_i)}{4}} dx - \log \int_{\infty}^{-C-\theta_i} \frac{1}{\sqrt{2\pi}} e^{\frac{-(x-\theta_i)}{4}} dx + \\ (R_i = r) \log \int_{C-\theta_i}^{\infty} \frac{1}{\sqrt{2\pi}} e^{\frac{-(x-\theta_i)}{4}} dx,\end{aligned}$$

where the indicator terms, $R_i = l, b, r$, are one if the response is respectively left, balance or right and zero otherwise.

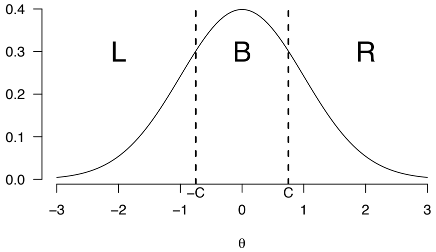

Figure A. The response probabilities expressed by the weighted-sum rule.

S1 Fig A shows a visual representation of these response probabilities. The set of functions is optimized with respect to α and C, using a constrained optimization implemented with the optim-function in Cran-R [41]. α is constrained between zero and one, and C higher than zero. Note that [13] also propose an implicit multiplication rule that can capture RIV. However it is not possible to estimate the parameters of this rule since the likelihood function is zero if α is zero, hence this rule will not be further studied.
