# Supplementary material for: The Balance-Scale Task Revisited: A Comparison of Statistical Models for Rule-Based and Information-Integration Theories of Proportional Reasoning
Source: PLoS One. 2015 Oct 27;10(10):e0136449. doi: 10.1371/journal.pone.0136449 (PMC4623502; doi:10.1371/journal.pone.0136449)
Supplement: S2 Text — (PDF) [file pone.0136449.s002.pdf]

S2 Text

S2 Table A shows the item description of the selected items of the Math Garden dataset. We also included W, CWA, CD, CB and multiple-pin items of the types B, W and D - for multiple pin items the weights are placed on two pins on each side of the fulcrum. Responses on these item types are not included in the results for this paper. Items where constructed such that the variation of the product-differences within the set of items of the same type is comparable between the different types.

Table A. Item Characteristics of the Math Garden and Paper-and-Pencil dataset

	Paper-and-Pencil						Math Garden					
Type	Wl	Dl	Wr	Dr	PD	Cor Resp	Wl	Dl	Wr	Dr	PD	Cor Resp
CBA	3	4	4	3	0	B	1	3	3	1	0	B
CBA	1	2	2	1	0	B	5	6	6	5	0	B
CBA	2	4	4	2	0	B	2	1	1	2	0	B
CBA	1	3	3	1	0	B	1	5	5	1	0	B
CDA	1	4	3	1	1	L	4	1	1	5	1	R
CDA	1	4	2	1	2	L	2	1	1	4	2	R
CDA	2	4	3	1	5	L	4	2	3	5	7	R
CDA	4	1	3	4	8	R	5	4	6	2	8	L
CW	1	3	2	2	1	R	3	3	1	6	3	L
CW	2	2	1	3	1	L	5	3	2	6	3	L
CW	3	3	2	4	1	R	2	5	1	6	4	L
CW	1	4	2	3	2	L	3	4	1	6	6	L
D	2	4	2	3	2	L	1	3	1	4	1	R
D	4	4	4	3	4	L	4	4	4	3	4	L
D	3	2	3	4	6	R	2	2	2	6	8	R
D	5	2	5	4	10	R	2	6	2	2	8	L

Note. The name of the item type refers to the dimension that determines the correct response; for example, in distance items, the beam goes down to the side with the largest distance between the peg with the weights and the fulcrum; Wl, Wr, Dl and Dr refer to respectively the number of blocks on the left and right side and the distance between the blocks and the fulcrum on the left and right side; PD = product-difference; Cor Resp = correct response.
