# Supplementary material for: The Balance-Scale Task Revisited: A Comparison of Statistical Models for Rule-Based and Information-Integration Theories of Proportional Reasoning
Source: PLoS One. 2015 Oct 27;10(10):e0136449. doi: 10.1371/journal.pone.0136449 (PMC4623502; doi:10.1371/journal.pone.0136449)
Supplement: S3 Text — (PDF) [file pone.0136449.s003.pdf]

S3 Text

Table A. Paper-and-Pencil: Fit Results LCM per Item Type

Type	Model	NC	BIC	1:p(BIC)	2:p(BIC)
D	Exploratory	2	2766	<.001	
		3	2661	.999	<.001
		4	2674	.001	
	Item Heterogeneity	3	2607		>.999
	Item Homogeneity	3(1)	2601		
		3(2)	2595		
		3(3)	2598		
	Final	3(1,2,3)	2581		
CW	Exploratory	2	4013	<.001	
		3	3899	>.999	<.001
		4	3934	<.001	
	Item Heterogeneity	3	3839		>.999
	Item Homogeneity	3(1)	3834		
		3(2)	3831		
		3(3)	3840		
	Final	3(1,2)	3826		
CDA	Exploratory	2	4047	<.001	
		3	3831	>.999	0.048
		4	3856	<.001	
	Item Heterogeneity	3	3825		0.952
	Item Homogeneity	3(1)	3837		
		3(2)	3864		
		3(3)	3925		
CBA	Exploratory	3	4280	<.001	
		4	4253	>.999	<.001
		5	4294	<.001	
	Item Heterogeneity	4	4233		>.999
	Item Homogeneity	4(1)	4221		
		4(2)	4224		
		4(3)	4240		
		4(4)	4227		
	Final	4(1,2,4)	4208		

Note. NC = the number of latent classes, the number between brackets refers to the class with constraints; p = BIC model probability of (1) select number of classes (2) select constraints; The best fitting model is underlined.

Table B. Math Garden: Fit Results LCM per Item Type

Type	Model	NC	BIC	1:p(BIC)	2:p(BIC)
WD	Exploratory	1	2839	<.001	
		2	2749	>.999	
		3	2784	<.001	
D	Exploratory	1	1330	0.018	
		2	1322	0.982	<.001
		3	1367	<.001	
	Item Heterogeneity	2	1303		>.999
	Item Homogeneity	2(1)	1293		
		2(2)	1304		
CW	Exploratory	1	4906	<.001	
		2	4785	0.999	0.165
		3	4798	0.001	
	Item Heterogeneity	2	4782		0.835
	Item Homogeneity	2(1)	4793		
		2(2)	4840		
CDA	Exploratory	1	3247	<.001	
		2	3209	0.992	0.003
		3	3219	0.008	
	Item Heterogeneity	2	3197		0.997
	Item Homogeneity	2(1)	3186		
		2(2)	3213		
CBA	Exploratory	1	4817	<.001	
		2	4763	0.920	>.999
		3	4768	0.080	
	Item Heterogeneity	2	4858		<.001
	Item Homogeneity	–	–		

Note. NC = the number of latent classes, the number between brackets refers to the class with constraints; p = BIC weights (1) select number of classes (2) select constraints; The best fitting model is underlined.
